# Pretransplant Fecal Carriage of Extended-Spectrum β-Lactamase–producing *Enterobacteriaceae* and Infection after Liver Transplant, France

**DOI:** 10.3201/eid1806.110139

**Published:** 2012-06

**Authors:** Frédéric Bert, Béatrice Larroque, Catherine Paugam-Burtz, Federica Dondero, François Durand, Estelle Marcon, Jacques Belghiti, Richard Moreau, Marie-Hélène Nicolas-Chanoine

**Affiliations:** Hôpital Beaujon, Clichy, France (F. Bert, B. Larroque, C. Paugam-Burtz, F. Dondero, F. Durand, E. Marcon, J. Belghiti, R. Moreau, M.H. Nicolas-Chanoine);; Université Paris VII Faculté de Médecine D. Diderot, Paris, France (C. Paugam-Burtz, F. Durand, J. Belghiti, M.H. Nicolas-Chanoine);; INSERM U 773 Centre de Recherche Biomédicale Bichat-Beaujon, CRB3, Paris (F. Durand, R. Moreau, M.H. Nicolas-Chanoine)

**Keywords:** liver transplantation, extended-spectrum β-lactamase, infection, fecal carriage, Enterobacteriaceae, transplant, liver, France, carbapenems, antimicrobial resistance, bacteria, ESBL

## Abstract

Bacterial infection after liver transplant is fairly common, mostly because liver transplant patients are severely ill and the surgery is very complex. Adding to the seriousness of this situation is that some bacteria are resistant to many antimicrobial drugs. However, treating all infections as drug resistant would lead to even more drug resistance, so only patients at highest risk should receive the most powerful drugs. But who is at highest risk? A recent study in France screened fecal samples of liver transplant candidates and found that post-operative infections were most likely for those patients who already had certain bacteria in their feces before surgery. Thus, fecal screening for those multiresistant bacteria should be considered for all liver transplant candidates so that if post-operative infection develops, those at high risk can receive the most specific drugs right away.

## Medscape CME Activity

Medscape, LLC is pleased to provide online continuing medical education (CME) for this journal article, allowing clinicians the opportunity to earn CME credit.

This activity has been planned and implemented in accordance with the Essential Areas and policies of the Accreditation Council for Continuing Medical Education through the joint sponsorship of Medscape, LLC and Emerging Infectious Diseases. Medscape, LLC is accredited by the ACCME to provide continuing medical education for physicians.

Medscape, LLC designates this Journal-based CME activity for a maximum of 1 *AMA PRA Category 1 Credit(s)*^TM^. Physicians should claim only the credit commensurate with the extent of their participation in the activity.

All other clinicians completing this activity will be issued a certificate of participation. To participate in this journal CME activity: (1) review the learning objectives and author disclosures; (2) study the education content; (3) take the post-test with a 70% minimum passing score and complete the evaluation at www.medscape.org/journal/eid; (4) view/print certificate.

**Release date: May 16, 2012; Expiration date: May 16, 2013**

## Learning Objectives

Upon completion of this activity, participants will be able to:

- Describe incidence and characteristics of ESBLE infections following liver transplantation, based on a 10-year study
- Describe clinical outcomes of ESBLE infections following liver transplantation, based on a 10-year study
- Describe the effect of ESBLE fecal carriage on risk for ESBLE infections following liver transplantation, and other risk factors for ESBLE infections following liver transplantation.

## CME Editor

**Shannon O’Connor,** Technical Writer/Editor, *Emerging Infectious Diseases. Disclosure: Shannon O’Connor has disclosed no relevant financial relationships.*

## CME Author

**Laurie Barclay, MD,** freelance writer and reviewer, Medscape, LLC. *Disclosure: Laurie Barclay, MD, has disclosed no relevant financial relationships.*

## Authors

*Disclosures:* ***Frédéric Bert, MD, PhD; Béatrice Larroque, MD, PhD; Federica Dondero, MD; Estelle Marcon; Jacques Belghiti, MD; Richard Moreau, MD;*** *and* ***Marie-Hélène Nicolas-Chanoine, MD, PhD,*** *have disclosed no relevant financial relationships.* ***Catherine Paugam-Burtz, MD, PhD,*** *has disclosed the following relevant financial relationships: served as a speaker or a member of a speakers bureau for Astellas.* ***François Durand, MD,*** *has disclosed the following relevant financial relationships: served as an advisor or consultant for Novartis, Astellas.*
