# Supplementary material for: Pretransplant Fecal Carriage of Extended-Spectrum β-Lactamase–producing Enterobacteriaceae and Infection after Liver Transplant, France
Source: Emerg Infect Dis. 2012 Jun;18(6):908–16. doi: 10.3201/eid1806.110139 (PMC3358139; doi:10.3201/eid1806.110139)
Supplement: Related texts

# Research article: Pretransplant Fecal Carriage of Extended-Spectrum β-Lactamase–producing *Enterobacteriaceae* and Infection after Liver Transplant, France

**DOI:** 10.3201/eid1806.110139

**Authors:** Frédéric Bert, Béatrice Larroque, Catherine Paugam-Burtz, Federica Dondero, François Durand, Estelle Marcon, Jacques Belghiti, Richard Moreau, Marie-Hélène Nicolas-Chanoine

**Affiliations:** Hôpital Beaujon, Clichy, France (F. Bert, B. Larroque, C. Paugam-Burtz, F. Dondero, F. Durand, E. Marcon, J. Belghiti, R. Moreau, M.H. Nicolas-Chanoine);; Université Paris VII Faculté de Médecine D. Diderot, Paris, France (C. Paugam-Burtz, F. Durand, J. Belghiti, M.H. Nicolas-Chanoine);; INSERM U 773 Centre de Recherche Biomédicale Bichat-Beaujon, CRB3, Paris (F. Durand, R. Moreau, M.H. Nicolas-Chanoine)

## Abstract

Extended-spectrum β-lactamase–producing *Enterobacteriaceae* isolates (ESBLE) are emerging pathogens that confer resistance to antimicrobial drugs. We conducted a 10-year study in France (January 2001–April 2010) to investigate the incidence of and risk factors for ESBLE infections after liver transplant. Of 710 transplant patients screened preoperatively for ESBLE fecal carriage, 5.5% had ESBLE infection develop within 4 months after surgery; patients with pretransplant ESBLE fecal carriage were more likely to have infection develop than were noncarriers. Typing showed extensive genetic diversity, with a large predominance of CTX-M enzymes. Independent predictors of ESBLE infection were pretransplant fecal carriage, Model for End Stage Liver Disease score >25, and return to surgery. Our results indicate that the influx of preoperatively acquired ESBLE isolates into the hospital outweighs cross-transmission in the epidemiology of ESBLE infections after liver transplant. Transplant candidates should be systematically screened for carriage, and posttransplant infection in carriers should be treated with carbapenems.

Extended-spectrum β-lactamase (ESBL)–producing *Enterobacteriaceae* (ESBLE) isolates, notably ESBL-producing *Escherichia coli* isolates, have emerged worldwide as a frequent cause of infection in hospitals and in the community (*1**,**2*). ESBLs confer resistance to all β-lactam agents except carbapenems and cephamycins and are frequently associated with resistance to other classes of antimicrobial drugs, including aminoglycosides and fluoroquinolones (*2**,**3*). Therapeutic options for infections caused by these multidrug-resistant organisms are therefore limited, which may result in delayed effective therapy and increased risk for death (*4**,**5*). Identifying risk factors for ESBLE infections enables identification of patients who should receive empirical treatment targeted to these organisms.

ESBLE infections may pose a particularly serious threat in profoundly immunosuppressed patients, such as transplant recipients, who are often empirically treated for postoperative infections. Solid organ transplant has been shown to be a risk factor for nosocomial ESBLE bacteremia (*6*), but little research has been conducted to identify risk factors for ESBLE infections among these patients, and previous studies included only kidney transplant recipients (*7**,**8*). Liver transplant (LT) recipients are particularly at risk for bacterial infections because of the severity of illness associated with preoperative end-stage liver disease and the technical complexity of surgery (*9*). After LT, bacterial infections occur in 35%–70% of patients; these infections are predominantly caused by *Enterobacteriaceae* (*10**–**13*). Although recent studies from Spain and China have reported alarmingly high rates of ESBL production in isolates from LT recipients (*14**–**16*), to our knowledge, the epidemiology of ESBLE infections among these high-risk patients in a nonepidemic setting has not been investigated.

We assessed the incidence of, risk factors for, and molecular epidemiology of ESBLE infections in LT recipients. More specifically, we investigated whether ESBLE pretransplant fecal carriage was an independent predictor of ESBLE infection after LT.

## Materials and Methods

### Study Population

An observational study of patients undergoing LT at Beaujon Hospital (Clichy, France) was conducted from January 1, 2001, through April 30, 2010. During this period, 734 patients underwent LT, and pretransplant screening for ESBLE fecal carriage was routinely performed as part of an infection control program. Rectal swab specimens were obtained preoperatively on the day of surgery from 710 of the 734 LT recipients; these 710 patients constituted the study population (Table 1).

**Table 1:** Characteristics of 710 liver transplant recipients included in study of ESBLE pretransplant fecal carriage and posttransplant infection, France, January 2001–April 2010*

Characteristic	Value
Age, y, mean ± SD	50 ± 11
Male sex	490 (69)
Viral hepatitis	261 (36.7)
Hepatocellular carcinoma	221 (31.1)
Alcoholism	196 (27.6)
Acute liver failure	74 (10.4)
Biliary disease	61 (8.6)
HIV infection	21 (3)
MELD score, mean ± SD	18 ± 9

*Values are no. (%) patients except as indicated. ESBLE, extended spectrum β-lactamase–producing *Enterobacteriaceae*; MELD, Model for End-stage Liver Disease.

During the study period, standard antimicrobial drug prophylaxis consisted of cefoxitin administered intraoperatively as described (*17*); primary immunosuppressive therapy consisted of tacrolimus, corticosteroids, and mycophenolate mofetil (*17*). Throughout the study period, isolation procedures were applied to patients infected or colonized with ESBLE isolates until they were discharged from the hospital. More specifically, these patients were placed in individual rooms, and contact isolation precautions, including the use of gowns and gloves, were implemented.

### Definition and Data Collection

ESBLE infections occurring within 4 months after LT were investigated. Infections were defined on the basis of clinical criteria and the isolation of an ESBLE isolate from a clinically significant site. Bacteremia was defined as a positive peripheral blood culture bottle result (*13**,**18*). Intraabdominal infection was considered to be present when an ESBLE isolate was cultured from biliary fluid, from peritoneal fluid containing >250 polymorphonuclear cells/mm^3^, or from an intraabdominal collection drained surgically or by percutaneous aspiration (*19*). Pneumonia was diagnosed on the basis of radiographic findings, clinical signs, and culture of >10^3^/mL of an ESBLE isolate from a protected distal aspirate (*20*). Urinary tract infection was diagnosed on the basis of urine leukocyte count and a positive culture >10^4^ cells/mL (*21*).

Data were collected in a prospectively maintained database. Preoperative data were age, gender, underlying liver disease, HIV infection, Model for End Stage Liver Disease (MELD) score, pretransplant ESBLE fecal carriage, stay in intensive care unit >48 h before surgery, hospitalization in the previous 6 months, and any history of bacteremia, spontaneous bacterial peritonitis, or both in the previous 6 months. Intraoperative variables were duration of surgery, cold ischemia time, combined kidney and liver transplant, type of donor (deceased or living), number of packed red blood cell units transfused, and type of biliary reconstruction (duct-to-duct or Roux-en-Y anastomosis). Postoperative data were retransplantation, return to surgery, and acute renal failure.

Outcome was assessed by death within 28 days after transplant, death while hospitalized, and death related to infection. Death was considered be related to infection when ESBL infection was the immediate cause of death or when it initiated a sequence of events leading to multiorgan failure and death.

### Microbiological Studies

A rectal swab specimen was routinely obtained before LT and plated onto a selective medium consisting of Drigalsky agar plates with 1 µg/mL cefotaxime during 2001–2004 and commercialized agar plates (bioMérieux, Marcy l’Etoile, France) during 2004–2010. Plates were incubated for 24–48 h. A single sample of each distinct type of colony growing on the selective media was picked and further studied. Isolates were identified to the species level by API 20E strips (bioMérieux), and ESBL production was confirmed by double disk synergy test (*22*). This test was performed by placing disks of cefotaxime, ceftazidime, cefepime and aztreonam on Mueller-Hinton agar at a distance of 30 mm, center to center, from an amoxicillin/clavulanate disk. In case of ambiguous results for ampicillin C producers, the presence of an ESBL was further investigated by a double disk synergy test with a disk spacing of 20 mm and another test performed on Mueller-Hinton agar containing cloxacillin (*22*).

*Enterobacteriaceae* isolates from clinical specimens were also identified by the API 20E strips, and antimicrobial drug susceptibility was tested by the Mueller-Hinton agar diffusion method and interpreted according to the recommendations of the Committee of the French Society for Microbiology (*23*). Clinical isolates were screened for ESBL production by the double-disk synergy test as described for rectal isolates. Rectal and clinical isolates from were kept frozen at −70°C until subcultured for strain and ESBL typing.

### Strain Typing

The clonal relatedness of ESBLE clinical isolates was studied by the enterobacterial repetitive intergenic consensus sequence type 2 (ERIC-2) PCR fingerprinting method (*24*). Electrophoretic banding patterns were compared by visual inspection. For patients who had ESBLE preoperative fecal carriage and subsequently had infection develop, the isolate from the rectal swab specimen and the isolate from the infected site were compared by using ERIC-2 PCR.

### β-Lactamase Typing

The main types of ESBL are SHV-, TEM-, and CTX-M ESBLs. These types were identified by using the Check-KPC ESBL microarray system (Check-Points, Wageningen, the Netherlands), according to the manufacturer’s instructions (*25**,**26*).

### Statistical Analysis

The prevalence of pretransplant ESBLE fecal carriage and subsequent ESBLE infections was compared among 4 periods (2001–2003, 2004–2006, 2007–2008, and 2009–2010) by a test for trend. Univariate analysis was used to identify associations between each of the preoperative, intraoperative, and postoperative variables and the occurrence of ESBLE infections. The χ^2^ test and, for small numbers, the Fisher exact test were used for comparison of categorical data. Continuous data were compared by Student *t* test; p<0.05 was considered significant. We performed stepwise logistic regression analysis to identify significant correlates of ESBLE infections: variables with a p value <0.1 were entered into the model. Potential interactions between covariates were considered. Data analyses were performed with SAS version 9.2 (SAS Institute, Inc., Cary, NC, USA).

## Results

### Preoperative ESBLE Fecal Carriage

Of the 710 patients screened preoperatively, 29 (4.1%) had ESBLE pretransplant fecal carriage; 4 patients each had 2 distinct ESBLE isolates. Species distribution of the 33 fecal isolates was as follows: *E. coli* (n = 21), *Enterobacter cloacae* (n = 5), *Klebsiella pneumoniae* (n = 4), *K. oxytoca* (n = 1), *Citrobacter koseri* (n = 1), and *C. freundii* (n = 1). The rate of pretransplant ESBLE fecal carriage increased significantly, from 0% to 10.6%, during the study period (Table 2).

**Table 2:** Temporal evolution of ESBLE pretransplant fecal carriage and posttransplant infection, France, by time period, January 2001–April 2010*

Variable	No. (%) patients				p value†
	2001–2003, n = 185	2004–2006, n = 196	2007–2008, n = 188	2009–2010, n = 141	
Pretransplant ESBLE fecal carriage	0	5 (2.5)	9 (4.8)	15 (10.6)	<0.0001
Posttransplant ESBLE infection	3 (1.6)	9 (4.6)	9 (4.8)	18 (12.8)	<0.0001

*ESBLE, extended spectrum β-lactamase–producing *Enterobacteriaceae*. †Comparison among the 4 periods by test of trend.

### ESBLE Infections

Of the 710 patients included in the study, 39 (5.5%) had ESBLE infection develop within 4 months after LT. The incidence rate of a first episode of ESBL infection was 5.1 cases/10,000 posttransplant days. The rate of infected patients increased significantly during the study period, from 1.6% during 2001–2003 to 12.8% during 2009–2010 (Table 2).

The median time from LT to ESBLE infection was 15 days (range 3–105 days). ESBLE infection was the first posttransplant infection in 15/39 patients. The sites of ESBL infection were as follows: intraabdominal (n = 19), urinary tract (n = 15), respiratory tract (n = 6), bloodstream (n = 2), and skin and soft tissues (n = 1). Eight patients had secondary bacteremia: 3 patients with peritonitis, 3 patients with cholangitis, 1 with pneumonia, and 1 with cellulitis. Overall, 10 (25.6%) of the 39 infected patients were bacteremic.

The predominant causative agent of infection in these patients was *E. coli* (n = 16), followed by *E. cloacae* (n = 11), *K. pneumoniae* (n = 5), *K. oxytoca* (n = 3), *C. koseri* (n = 2), and *Enterobacter aerogenes* (n = 2). The susceptibility rates of the 39 isolates are shown in Table 3. All isolates were susceptible to imipenem.

**Table 3:** Antimicrobial drug susceptibility, ERIC-2 PCR type, and β-lactamase gene content of the 39 clinical isolates of ESBL-producing *Enterobacteriaceae* identified, France, January 2001–April 2010*

Species	Year of isolation	Susceptibility				ERIC-2 PCR type	β-lactamase types†					
		TZP	FOX	AMK	CIP		CTX-M-1 group	CTX-M-9 group	ESBL TEM	Non-ESBL TEM	ESBL SHV	Non-ESBL SHV
*E. coli*	2003	S	S	S	R	1	+	–	–	+	–	–
*E. coli*	2004	R	S	S	R	2	+	–	–	+	–	–
*E. coli*	2004	R	R	S	S	3	+	–	–	–	–	–
*E. coli*	2007	R	R	R	R	4	+	–	–	–	–	–
*E. coli*	2007	S	S	S	R	5	+	–	–	+	–	+
*E. coli*	2008	S	S	R	R	6	+	–	–	–	–	–
*E. coli*	2008	R	S	S	R	7	+	–	–	–	–	–
*E. coli*	2008	S	S	S	S	8	+	–	–	+	–	+
*E. coli*	2009	S	S	R	R	9	+	–	–	+	–	–
*E. coli*	2009	S	S	S	R	10	–	+	–	–	–	–
*E. coli*	2009	S	R	R	R	9	+	–	–	+	–	–
*E. coli*	2009	R	S	R	R	11	+	+	–	+	–	–
*E. coli*	2009	R	S	S	R	4	+	–	–	+	–	–
*E. coli*	2010	R	S	R	R	12	+	–	–	–	–	–
*E. coli*	2010	S	S	S	S	13	+	–	–	–	–	–
*E. coli*	2010	R	R	R	R	14	+	–	–	–	–	–
*K. pneumoniae*	2008	R	S	S	R	15	+	–	–	–	–	+
*K. pneumoniae*	2008	R	S	S	R	16	+	–	–	–	–	+
*K. pneumoniae*	2010	R	R	R	R	17	+	–	–	+	–	+
*K. pneumoniae*	2010	R	R	R	R	17	+	–	–	+	–	+
*K. pneumoniae*	2010	R	S	S	R	15	+	–	–	–	+	–
*K. oxytoca*	2009	R	R	R	R	18	–	–	–	–	+	–
*K. oxytoca*	2009	R	R	R	R	18	–	–	–	–	+	–
*K. oxytoca*	2009	R	R	R	R	18	+	–	–	+	–	–
*C. koserii*	2006	S	R	R	R	19	–	+	+	–	–	–
*C. koserii*	2006	S	R	R	R	19	–	+	+	–	–	–
*E. cloacae*	2002	R	R	R	R	20	–	–	–	+	-	–
*E. cloacae*	2004	R	R	R	R	21	+	–	–	+	+	–
*E. cloacae*	2004	R	R	S	R	22	–	–	–	–	+	–
*E. cloacae*	2004	R	R	I	R	23	–	–	–	–	+	–
*E. cloacae*	2005	R	R	R	R	24	–	–	–	+	–	–
*E. cloacae*	2008	S	R	R	R	25	+	–	–	+	+	–
*E. cloacae*	2008	S	R	R	R	21	–	–	–	+	+	–
*E. cloacae*	2009	R	R	S	R	26	+	–	–	+	–	–
*E. cloacae*	2009	R	R	S	R	27	+	–	–	–	–	–
*E. cloacae*	2009	S	R	R	R	28	+	–	–	–	+	–
*E. cloacae*	2010	S	R	R	R	29	–	–	–	+	+	–
*E. aerogenes*	2002	R	R	R	R	30	+	–	+	–	–	–
*E. aerogenes*	2005	R	R	R	R	31	+	+	–	+	+	–

*ERIC-2, enterobacterial repetitive intergenic consensus sequence type 2; ESBL, extended-spectrum β-lactamase; TZP, piperacillin/tazobactam; FOX, cefoxitin; AMK, amikacin; CIP, ciprofloxacin; *E. coli*, *Escherichia coli*; S, susceptible; R, resistant*; K. pneumoniae*, *Klebsiella pneumoniae*; *K. oxytoca*, *Klebsiella oxytoca*; *C. koserii*, *Citrobacter koserii*; *E. cloacae*, *Enterobacter cloacae*; *E. aerogenes*, *Enterobacter aerogenes*. †The types of β-lactamase were identified by the Check-KPC ESBL microarray system (Check-Points, Wageningen, the Netherlands).

Six patients died within 28 days after ESBLE infection developed (28-day mortality rate 15.4%); 11 patients died during hospitalization (in-hospital mortality rate 28.2%). Death was related to the infection in 8 patients (infection-related mortality rate 20.5%). The in-hospital mortality rate was significantly higher in patients with ESBLE infection than in those without ESBLE infection (28.2% vs. 15.9%; p = 0.0455).

### Association between Fecal Carriage and Infection

ESBLE infection occurred in 13 (44.8%) of the 29 patients with pretransplant fecal carriage versus 26 (3.8%) of the 681 noncarriers (p<0.0001). Ten (84.6%) of the 13 patients with fecal carriage had ESBL infection develop within 14 days after LT. The median time to onset of infection was significantly shorter among patients with pretransplant fecal carriage than among those without carriage (9 days vs. 25 days; p = 0.0007). For the 13 carriers in whom ESBLE infection developed, the infecting isolate belonged to the same species as the fecal (or 1 of the fecal) isolate(s). In 10 of these 13 pairs of isolates, ERIC-2 PCR typing showed that the fecal isolate and the infecting isolate had identical patterns. An example of banding patterns is shown in the Figure.

**Figure:** Enterobacterial repetitive intergenic consensus sequence type 2 PCR patterns of pairs of *Escherichia coli* isolates from 6 patients examined during study of extended-spectrum β-lactamase–producing *Enterobacteriaceae* infection among liver transplant recipients, France, January 2001–April 2010. The pretransplant colonizing isolate (C) and the posttransplant infecting isolate (I) show identical patterns for patients 2, 3, and 6 and different patterns for patients 1, 4, and 5. M, molecular mass standard.

### Genetic Relatedness and Types of ESBLs

Overall, 31 ERIC-2 PCR profiles were observed among the 39 ESBLE clinical isolates (Table 3). Six of these patterns were observed in 2 isolates each (2 *E. coli*, 2 *K. pneumoniae*, 1 *E. cloacae*, 1 *C. koseri*), and 1 pattern was shared by the 3 *K. oxytoca* isolates. The remaining 24 patterns were observed in a single isolate each.

The Check-KPC ESBL microarray analysis (Check-Points) was used to identify the type(s) of ESBL in 37/39 tested isolates (Table 3). Nine of the isolates produced different types of ESBL, 8 isolates with 2 types and 1 isolate with 3 types; a single type of ESBL was detected in the remaining 28 isolates. Members of the CTX-M family, in particular those of the CTX-M-1 group, were most frequently detected (Table 3). Overall, enzymes of the CTX-M family were observed in 31 (79.5%) of the 39 isolates tested, including all *E. coli* isolates. The type of ESBL could not be identified in 2 isolates; this result may have resulted from plasmid loss, because the double-disk synergy test result was negative after subculture.

### Risk Factor Analysis

The characteristics of patients with and without ESBL infection are compared in Table 4. Variables significantly associated with infection in the univariate analysis were pretransplant ESBLE fecal carriage, acute liver failure, increased MELD score, prolonged hospitalization during the 6 months before LT, preoperative intensive care stay >48 h, return to surgery, and postoperative acute renal failure. The 10 variables with a p value <0.1 were entered in the stepwise logistic regression. In the final model, pretransplant ESBLE fecal carriage (odds ratio [OR] 18.4), MELD score >25 (OR 2.9), and return to surgery (OR 2.7) were independent predictors of ESBLE infection (Table 5). No significant interactions between the covariates kept in the model were found.

**Table 4:** Comparison of patients with and without ESBLE infection, by univariate analysis, France, January 2001–April 2010*

Variable	Patients with ESBLE infection, n = 39		Patients without ESBLE infection, n = 671	p value
Preoperative data				
Mean age, y	49		50	0.55
Male sex	24 (61.5)		466 (69.4)	0.30
ESBLE fecal carriage	13 (33.3)		16 (2.4)	<0.0001
Viral hepatitis	13 (33.3)		248 (37.1)	0.64
Hepatocellular carcinoma	7 (17.9)		214 (32.3)	0.061
Alcoholism	16 (41)		219 (32.6)	0.28
Acute liver failure	11 (28.2)		63 (9.4)	0.0011
HIV infection	1 (2.5)		20 (3)	1
MELD score >25	20 (51.3)		143 (21.4)	<0.0001
Intensive care stay >48 h†	14 (35.9)		80 (11.2)	<0.0001
Hospital stay >10 d†	20 (51.3)		185 (27.6)	0.0015
Spontaneous bacterial peritonitis†	7 (17.9)		63 (9.4)	0.082
Bacteremia†	5 (12.8)		70 (10.4)	0.59
Intraoperative data				
Mean duration of surgery, min	488		509	0.12
Mean cold ischemia time, min	472		484	0.47
Kidney transplant	2 (5.1)		39 (5.8)	1
Living donor	3 (7.9)		56 (8.4)	1
Mean no. units red blood cells transfused	5.9		4.5	0.11
Roux-en-Y anastomosis	4 (10.2)		46 (7.6)	0.53
Postoperative data				
Retransplantation		3 (7.7)	40 (6)	0.72
Return to surgery		23 (59)	228 (34)	0.0015
Acute renal failure		24 (66.7)	238 (38.1)	0.0007

*Values are no. (%) except as indicated. ESBLE, extended-spectrum β-lactamase–producing *Enterobacteriaceae*; MELD, Model for End-stage Liver Disease. †In the 6 mo before liver transplant.

**Table 5:** Risk factors for ESBLE infection after liver transplant, by stepwise logistic regression analysis, France, January 2001–April 2010*

Variable†	OR (95% CI)	p value
Pretransplant ESBLE fecal carriage	18.4 (7.1–47.5)	<0.0001
MELD score >25	2.9 (1.4–6.2)	0.0053
Return to surgery	2.7 (1.3–5.9)	0.011

*ESBLE, extended-spectrum β-lactamase-producing *Enterobacteriaceae*; OR, odds ratio; MELD, Model for End-stage Liver Disease. †Other factors initially in the model were hepatocellular carcinoma, acute liver failure, pretransplant ICU stay >48h, hospital stay >10 d in the 6 mo before transplant, spontaneous bacterial peritonitis in the 6 mo before transplant, red blood cells transfused, and posttransplant acute renal failure.

## Discussion

We demonstrated a progressive and significant increase in the incidence of pretransplant ESBLE fecal carriage and posttransplant infection among LT patients in our transplant center over the past decade. We also identified pretransplant fecal carriage as an independent risk factor for subsequent ESBLE infection. Lastly, we found high clonal diversity and a vast majority of CTX-M-1 group and CTX-M-9 group enzymes among our isolates.

Recent studies suggest that nonhospitalized patients are increasingly important reservoirs of ESBLE isolates; these community reservoirs may play a role in the epidemiology of ESBLE infections in hospital settings (*27**–**30*). Harris et al. reported that 2% of patients admitted to intensive care were colonized by an ESBL-producing *E. coli* or *Klebsiella* spp. isolate (*31*). In 2 other recent studies, the rates of ESBLE fecal carriage at hospital admission were 10.8% and 8%, respectively (*29**,**32*). Overall, the rate of pretransplant carriage was 4.1% in our liver transplant recipients, and a significant increase, from 0% to 10.6%, was noted over the study period. These isolates may have been acquired in the community or during previous hospitalizations.

Overall, 5.5% of patients in our study had ESBLE infection develop within 4 months after LT. This infection rate increased in similar proportions to the pretransplant colonization rate over time, reaching 12.8% during 2009–2010. We found a significant association between fecal colonization status and occurrence of subsequent infection. Posttransplant ESBLE infection developed in ≈45% of carriers; most infections occurred within 14 days after LT, and molecular typing showed that the infecting isolate was identical to the isolate from the prettransplant fecal swab for most of these patients. This finding is consistent with previous studies in nontransplant patients that found prior colonization with an ESBLE isolate confers an increased risk for hospital-acquired infection with the same organism (*29**,**33**,**34*). In contrast, Arnan et al. recently found that fecal carriage of ESBL-producing *E. coli* was not associated with infection in neutropenic patients who had hematologic malignancies (*35*).

Differences in infection rates on the basis of pretransplant colonization status may indicate that fecal carriage plays a direct role in the pathogenesis of ESBLE infection or may only reflect differences in preoperative host characteristics. In this study, we used a multivariate logistic regression analysis to control for the effects of confounding variables such as the severity of underlying liver disease. The results indicate that pretransplant fecal carriage is an independent risk factor for ESBLE infection after LT. Other predictors of infection in the multivariate analysis were high preoperative MELD score and return to surgery, 2 factors previously identified as risk factors for *Enterobacteriaceae* bacteremia in LT recipients (*18*).

Molecular typing of our isolates showed extensive genetic diversity. This finding strongly suggests that in-hospital spread of specific clones was not the cause of the increase in the frequency of ESBLE infections in our transplant center. An active infection-control program for multidrug-resistant organisms was in place during the study period, including isolation procedures for preventing patient-to-patient transmission of ESBLE isolates. Similarly, Harris et al. reported that cross-transmission was not an important cause of acquisition of ESBL-producing *E. coli* in the intensive care unit setting (*36*). However, it is noteworthy that ESBLE infection occurred in 26 patients whose pretransplant fecal carriage screening tests were negative, which suggests postoperative acquisition. An alternative explanation is that rectal screening may have failed to detect colonization in patients with a low intestinal inoculum.

Prior exposure to antimicrobial drugs is a well-defined risk factor for ESBLE colonization and infection in hospitalized patients and in the community (*6**,**29**–**33**,**35**,**37*). Patients undergoing LT are particularly likely to be exposed to antimicrobial drugs, but this exposure is difficult to evaluate because all patients receive intraoperative prophylaxis, and many receive antimicrobial drugs in the early postoperative period for suspected or documented bacterial infections. In this study, ESBLE infection was not the first postoperative infection in 24/39 infected patients, which indicates previous antimicrobial drug use after LT. Moreover, antimicrobial drugs given for pretransplant infections, which occur in 30%–35% of candidates for LT (*38*), may have predisposed them to ESBLE colonization and subsequent infection. Pretransplant antimicrobial drug exposure also includes norfloxacin prophylaxis in patients with a history of spontaneous bacterial peritonitis. Unfortunately, we did not have access to information about all antimicrobial drugs that patients may have received as outpatients or during hospitalizations in other institutions during the waiting period. Further studies are therefore needed to identify predictors of pretransplant ESBLE fecal carriage among LT recipients.

Our results have implications for the treatment of infections in LT recipients. Because ESBLE infections may be associated with inappropriate initial therapy and increased risk for death, the identification of high-risk patients is useful when choosing empiric antimicrobial drugs treatment. Therefore, we suggest that candidates for LT be systematically screened for ESBLE fecal carriage. Carbapenems should be included in initial empiric therapy for infections in LT recipients who have documented ESBLE fecal carriage.

The main limitation of our work is that it was a single-center study, which means the results may not be applicable to settings with a different epidemiologic context. However, our center is one of the largest LT centers in France and receives referrals from throughout the country. Moreover, our findings are consistent with most recent reports on the epidemiology of ESBLE infections worldwide. During the past decade, *E. coli* has become the most prevalent species among ESBLE isolates, and CTX-M enzymes have replaced TEM and SHV enzymes as the most common types of ESBLs (*1**,**2*). Similarly, *E. coli* was the most frequent species identified among our isolates, and ESBL typing revealed a large predominance of CTX-M-1 group enzymes. Therefore, we assume our findings can be generalized to other LT centers that have similar infection control strategies. Another limitation of our study is that some ESBLE isolates may have been unidentified in patients carrying different strains of ESBL-producing *E. coli*.

In summary, preoperative fecal carriage is an independent predictor of ESBLE infection after LT. The influx of preoperatively acquired ESBLE isolates into the hospital outweighs posttransplant cross-transmission in the epidemiology of these infections. Given the worldwide ongoing diffusion of ESBLE isolates outside hospitals, the proportion of LT recipients in whom ESBLE infections develop is likely to further increase over time, in spite of adequate infection control measures. Therefore, these multidrug-resistant organisms may become a major threat in LT centers in the near future.

## References

[1] Cantón R, Coque TM. The CTX-M β-lactamase pandemic. Curr Opin Microbiol. 2006;9:466–75. 10.1016/j.mib.2006.08.01116942899

[2] Pitout JD, Laupland KB. Extended-spectrum β-lactamase–producing *Enterobacteriaceae*: an emerging public-health problem. Lancet Infect Dis. 2008;8:159–66. 10.1016/S1473-3099(08)70041-018291338

[3] Schwaber MJ, Navon-Venezia S, Schwartz D, Carmeli Y. High levels of antimicrobial coresistance among extended-spectrum-β-lactamase–producing *Enterobacteriaceae.* Antimicrob Agents Chemother. 2006;50:1257–62. 10.1128/AAC.50.4.1257-1262.200615855548PMC1087677

[4] Schwaber MJ, Carmeli Y. Mortality and delay in effective therapy associated with extended-spectrum β-lactamase production in *Enterobacteriaceae* bacteremia: a systematic review and meta-analysis. J Antimicrob Chemother. 2007;60:913–20. 10.1093/jac/dkm31817848376

[5] Tumbarello M, Sanguinetti M, Montuori E, Trecarichi EM, Posteraro B, Fiori B, Predictors of mortality in patients with bloodstream infections caused by extended-spectrum β-lactamase–producing *Enterobacteriaceae*: importance of inadequate initial antimicrobial treatment. Antimicrob Agents Chemother. 2007;51:1987–94. 10.1128/AAC.01509-0617387156PMC1891412

[6] Rodríguez-Baño J, Picón E, Gijón P, Hernández JR, Cisneros JM, Peña C, Risk factors and prognosis of nosocomial bloodstream infections caused by extended-spectrum β-lactamase–producing *Escherichia coli.* J Clin Microbiol. 2010;48:1726–31. 10.1128/JCM.02353-0920181897PMC2863889

[7] Linares L, Cervera C, Cofán F, Lizaso D, Marco F, Ricart MJ, Risk factors for infection with extended-spectrum and ampC β-lactamase–producing Gram-negative rods in renal transplantation. Am J Transplant. 2008;8:1000–5. 10.1111/j.1600-6143.2008.02197.x18727176

[8] Pinheiro HS, Mituiassu AM, Carminatti M, Braga AM, Bastos MG. Urinary tract infection caused by extended-spectrum beta-lactamase–producing bacteria in kidney transplant patients. Transplant Proc. 2010;42:486–7. 10.1016/j.transproceed.2010.02.00220304172

[9] Rubin RH. Infectious disease problems. In: Maddrey WC, Schiff ER, Sorell MF, editors. Transplantation of the liver. Philadelphia: Lippincott Williams & Wilkins; 2001. p. 275–295.

[10] George DL, Arnow PM, Fox AS, Baker AL, Thistlethwaite JR, Emond JC, Bacterial infection as a complication of liver transplantation: epidemiology and risk factors. Rev Infect Dis. 1991;13:387–96. 10.1093/clinids/13.3.3871866541

[11] Wade JJ, Rolando N, Hayllar K, Philpott-Howard J, Casewell MW, Williams R. Bacterial and fungal infections after liver transplantation: an analysis of 284 patients. Hepatology. 1995;21:1328–36. 10.1002/hep.18402105177737639

[12] Singh N, Wagener MM, Obman A, Cacciarelli TV, de Vera ME, Gayowski T. Bacteremias in liver transplant recipients: shift toward Gram-negative bacteria as predominant pathogens. Liver Transpl. 2004;10:844–9. 10.1002/lt.2021415237367

[13] Bert F, Larroque B, Paugam-Burtz C, Janny S, Durand F, Dondero F, Microbial epidemiology and outcome of bloodstream infections in liver transplant recipients: an analysis of 259 episodes. Liver Transpl. 2010;16:393–401.2020959810.1002/lt.21991

[14] García Prado ME, Matia EC, Ciuro FP, Díez-Canedo JS, Sousa Martín JM, Porras López FM, Surgical site infection in liver transplant recipients: impact of the type of perioperative prophylaxis. Transplantation. 2008;85:1849–54. 10.1097/TP.0b013e318173540718580480

[15] Zhou JD, Guo JJ, Zhang Q, Chen Y, Zhu SH, Peng HY. Drug resistance of infectious pathogens after liver transplantation. Hepatobiliary Pancreat Dis Int. 2006;5:190–4.16698573

[16] Shi SH, Kong HS, Xu J, Zhang WJ, Jia CK, Wang WL, Multidrug resistant gram-negative bacilli as predominant bacteremic pathogens in liver transplant recipients. Transpl Infect Dis. 2009;11:405–12. 10.1111/j.1399-3062.2009.00421.x19638006

[17] Bert F, Bellier C, Lassel L, Lefranc V, Durand F, Belghiti J, Risk factors for *Staphylococcus aureus* infection in liver transplant recipients. Liver Transpl. 2005;11:1093–9. 10.1002/lt.2049116123951

[18] Bellier C, Bert F, Durand F, Retout S, Belghiti J, Mentré F, Risk factors for *Enterobacteriaceae* bacteremia after liver transplantation. Transpl Int. 2008;21:755–63. 10.1111/j.1432-2277.2008.00673.x18435684

[19] Reid GE, Grim SA, Sankary H, Benedetti E, Oberholzer J, Clark NM. Early intra-abdominal infections associated with orthotopic liver transplantation. Transplantation. 2009;87:1706–11. 10.1097/TP.0b013e3181a6033819502964

[20] Weiss E, Dahmani S, Bert F, Janny S, Sommacale D, Dondero F, Early-onset pneumonia after liver transplantation: microbiological findings and therapeutic consequences. Liver Transpl. 2010;16:1178–85. 10.1002/lt.2213220879016

[21] Horan TC, Gaynes RP. Surveillance of nosocomial infections. In: Mayhall CG, editor. Hospital epidemiology and infection control, 3rd ed. Baltimore: Lippincott Williams & Wilkins; 2004. p. 1659–702.

[22] Garrec H, Drieux-Rouzet L, Golmard J-L, Jarlier V, Robert J. Comparison of nine phenotypic methods for detection of extended-spectrum β-lactamase production by *Enterobacteriaceae.* J Clin Microbiol. 2011;49:1048–57. 10.1128/JCM.02130-1021248086PMC3067698

[23] Communiqué de la Société Française de Microbiologie [cited 2011 Aug 22]. http://www.sfm-microbiologie.org/pages/?page=746&id_page=182.

[24] Bert F, Johnson JR, Ouattara B, Leflon-Guibout V, Johnston B, Marcon E, Genetic diversity and virulence profile of *Escherichia coli* isolates causing spontaneous bacterial peritonitis and bacteremia in patients with cirrhosis. J Clin Microbiol. 2010;48:2709–14. 10.1128/JCM.00516-1020519468PMC2916625

[25] Endimiani A, Hujer AM, Hujer KM, Gatta JA, Schriver AC, Jacobs MR, Evaluation of a commercial microarray system for detection of SHV-, TEM-, CTX-M, and KPC-type β-lactamase genes in Gram-negative isolates. J Clin Microbiol. 2010;48:2618–22. 10.1128/JCM.00568-1020504993PMC2897517

[26] Naas T, Cuzon G, Truong H, Bernabeu S, Nordmann P. Evaluation of a DNA microarray, the Check-Points ESBL/KPC array, for rapid detection of TEM, SHV, and CTX-M extended-spectrum β-lactamases and KPC carbapenemases. Antimicrob Agents Chemother. 2010;54:3086–92. 10.1128/AAC.01298-0920547813PMC2916358

[27] Valverde A, Coque TM, Sánchez-Moreno MP, Rollán A, Baquero F, Cantón R. Dramatic increase in prevalence of fecal carriage of extended-spectrum β-lactamase–producing *Enterobacteriaceae* during nonoutbreak situations in Spain. J Clin Microbiol. 2004;42:4769–75. 10.1128/JCM.42.10.4769-4775.200415472339PMC522353

[28] Rodríguez-Baño J, Alcalá JC, Cisneros JM, Grill F, Oliver A, Horcajada JP, Community infections caused by extended-spectrum β-lactamase–producing *Escherichia coli.* Arch Intern Med. 2008;168:1897–902. 10.1001/archinte.168.17.189718809817

[29] Ben-Ami R, Schwaber MJ, Navon-Venezia S, Schwartz D, Giladi M, Chmelnitsky I, Influx of extended-spectrum β-lactamase–producing *Enterobacteriaceae* into the hospital. Clin Infect Dis. 2006;42:925–34. 10.1086/50093616511754

[30] Ben-Ami R, Rodríguez-Baño J, Arslan H, Pitout JDD, Quentin C, Calbo ES, A multinational survey of risk factors for infection with extended-spectrum β-lactamase producing *Enterobacteriaceae* in nonhospitalized patients. Clin Infect Dis. 2009;49:682–90. 10.1086/60471319622043

[31] Harris AD, McGregor JC, Johnson JA, Strauss SM, Moore AC, Standiford HC, Risk factors for colonization with extended-spectrum β-lactamase–producing bacteria and intensive care unit admission. Emerg Infect Dis. 2007;13:1144–9.1795308310.3201/eid1308.070071PMC2828082

[32] Friedmann R, Raveh D, Zartzer E, Rudensky B, Broide E, Attias D, Prospective evaluation of colonization with extended-spectrum β-lactamase (ESBL) -producing Enterobacteriaceae among patients at hospital admission and of subsequent colonization with ESBL-producing *Enterobacteriaceae* among patients during hospitalization. Infect Control Hosp Epidemiol. 2009;30:534–42. 10.1086/59750519419270

[33] Martins IS, Pessoa-Silva CL, Nouer SA, Pessoa de Araujo EG, Ferreira AL, Riley LW, Endemic extended-spectrum β-lactamase–producing *Klebsiella pneumoniae* at an intensive care unit: risk factors for colonization and infection. Microb Drug Resist. 2006;12:50–8. 10.1089/mdr.2006.12.5016584309

[34] Reddy P, Malczynski M, Obias A, Reiner S, Jin N, Huang J, Screening for extended-spectrum β-lactamase producing *Enterobacteriaceae* among high-risk patients and rates of subsequent bacteremia. Clin Infect Dis. 2007;45:846–52. 10.1086/52126017806048

[35] Arnan M, Gudiol C, Calatayud L, Liñares J, Dominguez MÁ, Ribera JM, Risk factors for, and clinical relevance of, faecal extended-spectrum β-lactamase producing *Escherichia coli* (ESBLEC) carriage in neutropenic patients with haematological malignancies. Eur J Clin Microbiol Infect Dis. 2011;30:355–60. 10.1007/s10096-010-1093-x21052757

[36] Harris AD, Kotetishvili M, Shurland S, Johnson JA, Morris JG, Nemoy LL, How important is patient-to-patient transmission in extended-spectrum beta-lactamase *Escherichia coli* acquisition. Am J Infect Control. 2007;35:97–101. 10.1016/j.ajic.2006.09.01117327188

[37] Peña C, Gudiol C, Tubau F, Saballs M, Pujol M, Dominguez MA, Risk-factors for acquisition of extended-spectrum β-lactamase–producing *Escherichia coli* among hospitalised patients. Clin Microbiol Infect. 2006;12:279–84. 10.1111/j.1469-0691.2005.01358.x16451416

[38] Sun H-Y, Cacciarelli TV, Singh N. Impact of pretransplant infections on clinical outcomes of liver transplant recipients. Liver Transpl. 2010;16:222–8. 10.1002/lt.2198220104499

# Research article: Pretransplant Fecal Carriage of Extended-Spectrum β-Lactamase–producing *Enterobacteriaceae* and Infection after Liver Transplant, France

**DOI:** 10.3201/eid1806.110139

**Authors:** Frédéric Bert, Béatrice Larroque, Catherine Paugam-Burtz, Federica Dondero, François Durand, Estelle Marcon, Jacques Belghiti, Richard Moreau, Marie-Hélène Nicolas-Chanoine

**Affiliations:** Hôpital Beaujon, Clichy, France (F. Bert, B. Larroque, C. Paugam-Burtz, F. Dondero, F. Durand, E. Marcon, J. Belghiti, R. Moreau, M.H. Nicolas-Chanoine);; Université Paris VII Faculté de Médecine D. Diderot, Paris, France (C. Paugam-Burtz, F. Durand, J. Belghiti, M.H. Nicolas-Chanoine);; INSERM U 773 Centre de Recherche Biomédicale Bichat-Beaujon, CRB3, Paris (F. Durand, R. Moreau, M.H. Nicolas-Chanoine)

## Medscape CME Quiz

## Earning CME Credit

To obtain credit, you should first read the journal article. After reading the article, you should be able to answer the following, related, multiple-choice questions. To complete the questions (with a minimum 70% passing score) and earn continuing medical education (CME) credit, please go to www.medscape.org/journal/eid. Credit cannot be obtained for tests completed on paper, although you may use the worksheet below to keep a record of your answers. You must be a registered user on Medscape.org. If you are not registered on Medscape.org, please click on the New Users: Free Registration link on the left hand side of the website to register. Only one answer is correct for each question. Once you successfully answer all post-test questions you will be able to view and/or print your certificate. For questions regarding the content of this activity, contact the accredited provider, CME@medscape.net. For technical assistance, contact CME@webmd.net. American Medical Association’s Physician’s Recognition Award (AMA PRA) credits are accepted in the US as evidence of participation in CME activities. For further information on this award, please refer to http://www.ama-assn.org/ama/pub/category/2922.html. The AMA has determined that physicians not licensed in the US who participate in this CME activity are eligible for *AMA PRA Category 1 Credits*™. Through agreements that the AMA has made with agencies in some countries, AMA PRA credit may be acceptable as evidence of participation in CME activities. If you are not licensed in the US, please complete the questions online, print the certificate and present it to your national medical association for review.

## Article Title: Pretransplant Fecal Carriage of Extended-Spectrum β-Lactamase–producing *Enterobacteriaceae* and Infection after Liver Transplant, France

## CME Questions

**1. Your patient is a 62-year-old male about to undergo liver transplantation. Based on the 10-year study by Dr. Bert and colleagues, which of the following statements about incidence and characteristics of extended-spectrum β-lactamase–producing *Enterobacteriaceae* (ESBLE) infections following liver transplantation is most likely correct?**

A. Incidence of ESBLE infection within 4 months of surgery remained stable over the study period

B. During the study period, overall incidence of ESBLE infection within 4 months of liver transplantation was 5.5%

C. *E. cloacae* was the predominant causative agent

D. The median time from liver transplantation to ESBLE infection was 5 days

**2. The patient described in question 1 develops ESBLE infection after liver transplantation. Based on the 10-year study by Dr. Bert and colleagues, which of the following statements about clinical outcomes of ESBLE infection is most likely correct?**

A. Respiratory tract is the most likely site of ESBLE infection

B. Bacteremia occurs in about half of patients with ESBLE infection

C. In-hospital mortality rate is about 28%

D. About one quarter of ESBLE infections are resistant to imipenem

**3. Based on the 10-year study by Dr. Bert and colleagues, which of the following statements about risk factors for ESBLE infections following liver transplantation would most likely be correct?**

A. About one quarter of patients with ESBLE pretransplant fecal carriage developed ESBLE infections following liver transplantation

B. Independent predictors of ESBLE infections following liver transplantation were pretransplant ESBLE fecal carriage, MELD score ≥25, and return to surgery

C. Risk of ESBLE infections following liver transplantation was 5-fold higher in patients with pretransplant ESBLE fecal carriage

D. In about half of patients with pretransplant ESBLE fecal carriage and posttransplant ESBLE infection, the rectal isolate and the infecting isolate had identical patterns on ERIC-2 PCR typing

## Activity Evaluation

**Table:** 

1. The activity supported the learning objectives.				
Strongly Disagree				Strongly Agree
1	2	3	4	5
2. The material was organized clearly for learning to occur.				
Strongly Disagree				Strongly Agree
1	2	3	4	5
3. The content learned from this activity will impact my practice.				
Strongly Disagree				Strongly Agree
1	2	3	4	5
4. The activity was presented objectively and free of commercial bias.				
Strongly Disagree				Strongly Agree
1	2	3	4	5
